# Barriers to and Facilitators of Automated Patient Self-scheduling for Health Care Organizations: Scoping Review

**DOI:** 10.2196/28323

**Published:** 2022-01-11

**Authors:** Elizabeth W Woodcock

**Affiliations:** 1 Department of Health Policy & Management Rollins School of Public Health Emory University Atlanta, GA United States

**Keywords:** appointment, scheduling, outpatient, ambulatory, online, self-serve, e-book, web-based, automation, patient satisfaction, self-scheduling, eHealth, digital health, mobile phone

## Abstract

**Background:**

Appointment management in the outpatient setting is important for health care organizations, as waits and delays lead to poor outcomes. Automated patient self-scheduling of outpatient appointments has demonstrable advantages in the form of patients’ arrival rates, labor savings, patient satisfaction, and more. Despite evidence of the potential benefits of self-scheduling, the organizational uptake of self-scheduling in health care has been limited.

**Objective:**

The objective of this scoping review is to identify and to catalog existing evidence of the barriers to and facilitators of self-scheduling for health care organizations.

**Methods:**

A scoping review was conducted by searching 4 databases (PubMed, CINAHL, Business Source Ultimate, and Scopus) and systematically reviewing peer-reviewed studies. The Consolidated Framework for Implementation Research was used to catalog the studies.

**Results:**

In total, 30 full-text articles were included in this review. The results demonstrated that self-scheduling initiatives have increased over time, indicating the broadening appeal of self-scheduling. The body of literature regarding intervention characteristics is appreciable. Outer setting factors, including national policy, competition, and the response to patients’ needs and technology access, have played an increasing role in influencing implementation over time. Self-scheduling, compared with using the telephone to schedule an appointment, was most often cited as a relative advantage. Scholarly pursuit lacked recommendations related to the framework’s inner setting, characteristics of individuals, and processes as determinants of implementation. Future discoveries regarding these Consolidated Framework for Implementation Research domains may help detect, categorize, and appreciate organizational-level barriers to and facilitators of self-scheduling to advance knowledge regarding this solution.

**Conclusions:**

This scoping review cataloged evidence of the existence, advantages, and intervention characteristics of patient self-scheduling. Automated self-scheduling may offer a solution to health care organizations striving to positively affect access. Gaps in knowledge regarding the uptake of self-scheduling by health care organizations were identified to inform future research.

## Introduction

### Background

Appointment management in the outpatient setting is important for health care organizations, as waits and delays lead to poor outcomes. The Institute of Medicine has 6 aims for health care organizations to improve quality [1]. Despite the goal of timely access to care, the topic of visit timeliness is one of the least evaluated and understood aspects of care delivery, and there is little assessment of what drives care timeliness and the potential approaches for improving this dimension of care [2]. Appointment wait times and scheduling difficulties can negatively affect patient satisfaction [3-5], access to care [6], patient safety [7], and health care use and organizational reputation [2]. Timely access has a broader impact on the delivery of cost-effective health care [8] and individuals’ well-being [9]. The association between patient experience and the perception of quality of care has been demonstrated by Schneider et al [10]. Reasonable wait times are expected by patients [11,12].

Outside of health care, other industries with limited resources have addressed timeliness to service by engaging customers through self-service. For example, the transportation and hospitality industries have experienced improvements in operations [13,14], profitability [15], customer loyalty [16], and customer wait times [17] via the execution of consumer-based reservation systems. At present, consumers make reservations for services from a multitude of non–health care businesses. However, the adoption of management technologies, such as the self-scheduling of appointments in health care, has trailed other industries.

### Benefits

There is evidence that automated self-scheduling provides value and that health care organizations can benefit from it. Researchers have identified the advantages of automated patient self-scheduling for health care organizations in the form of labor savings [18-22], information transparency [23,24], cost reduction [25], cycle time [26], patient satisfaction [27,28], patient accountability [29], patient information [30], patient time savings [31], physician punctuality [32], patient loyalty [23], and patient attendance [33-37]. Reducing missed appointments increases a health care organization’s efficiency and the effective allocation of resources [38]. Automated self-scheduling eliminates the barriers inherent in the fixed capacity of phone lines and scheduling staff [39].

Health care organizations are faced with the need to increase access to accommodate patients’ changing expectations [40,41]. Self-scheduling may offer the convenience that patients seek [42,43]. Countries in Europe [44], England [19,34], Canada [36], Australia [45], and the United States [23], have established health technology initiatives at the national level. Nigeria [20], India [30], Taiwan [22], the Philippines [26], and Iraq and the Kurdistan region [46] have determined that self-scheduling may serve as a better alternative to obtaining an appointment as opposed to the traditional process of accessing outpatient care by physically standing in line. In Iran [47] and China [24,48,49], hospitals are mandated to provide the capability, in part, to address the problems associated with in-person queues for appointments. In Estonia, this functionality is built into the national system [50]. The benefits of self-scheduling may not be realized by persons in low- and middle-income countries, where many patients report negative experiences related to poor communication, short visits, or lengthy waits [51]. Self-scheduling may be perceived as elusive or ineffective, with patients preferring to physically wait in line to combat inefficiencies. This may not be a malfunction of the technological solution but rather a result of low- and middle-income countries’ failure to address socioeconomic disparities that have eroded patients’ confidence in the health care system [52].

### Adoption

Despite evidence of the potential benefits of self-scheduling, the organizational uptake of self-scheduling in health care has been limited. The lack of adoption may be a result of several factors examined in other studies of technology adoption, including the absence of financial incentives for the organization [53], cost [54], leadership [55], and policy and regulations [56]. Health care providers have expressed reluctance about self-scheduling based on cost, flexibility, safety, and integrity; patients cited concerns based on their prior experience with computers and the internet, as well as communication preferences [21]. Organizations may be reacting to patient hesitancy. Despite the infusion of technology in daily living, patients exhibit reluctance to automation in health care, citing concerns about accuracy, security, and the lack of empathy compared with human interactions [57].

There is a small body of literature regarding organizational barriers to the adoption of automated self-scheduling in popular literature. A practicing physician, informaticist, and the founder of a software company that offered self-scheduling products, Dr Jonathan Teich, revealed the following to the American Medical News in 2004 [58]:

Before you can successfully implement self-scheduling, you have to implement “Mabel.” Mabel is the generic scheduling administrator who has been working for Dr. Smith for 35 years, and knows a thousand nuances and idiosyncrasies and preferences that have been silently established over the years...Unfortunately for the computer world, it’s extremely difficult to find out what Mabel really knows, let alone try and put it into an algorithm.

Research has demonstrated that physicians’ concerns about addressing scheduling complexity [58] and preferences [59] are key factors in scheduling, with physicians expressing a fear of losing control of their schedules [60-62].

A previous review in this field provided evidence of facilitators of (no-shows, labor, waiting time, and patient satisfaction) and barriers to (cost, flexibility, safety, and integrity) automated self-scheduling [21]. Patients’ expectations regarding their health care experience, as well as the application, adoption, and use of health care technology have evolved significantly since the publication of the systematic review in 2017, thereby compelling a new review to be performed.

### Aim

Against this background, this scoping review seeks to identify the barriers to and facilitators of self-scheduling for health care organizations. The scoping review technique was selected based on a broad research question, the pursuit of identifying content without judging the quality of the material, and the intention to perform a qualitative synthesis [63].

## Methods

The five-step process for scoping reviews by Arksey and O’Malley [64] was deployed for this study: (1) identification of the research question; (2) identification of relevant studies; (3) study selection; (4) charting the data; and (5) collating, summarizing, and reporting the results.

### Step 1: Identification of the Research Question

The following research questions guided the review: What are the barriers to and facilitators of health care organizations’ uptake of automated patient self-scheduling? What are the gaps in the literature regarding barriers and facilitators?

### Step 2: Identification of Relevant Studies

This scoping review was performed by searching electronic databases according to the PRISMA-S (Preferred Reporting Items for Systematic Reviews and Meta-Analyses Search) guidelines [65]. The databases used were PubMed, CINAHL, Business Source Ultimate, and Scopus. The search strategy was developed with the assistance of an informaticist specializing in reviews. The search terms for self-scheduling were developed by researching titles, keywords, and commonly used phrases in the relevant literature. The search strategy was initiated on PubMed using combinations and word variations of key terms for the scoping review: “self-scheduling,” “automated scheduling,” “Web-based scheduling,” “e-appointments,” “online scheduling,” “Internet scheduling,” and “self-serve scheduling.” Additional terms were integrated using keywords from articles of interest that were retrieved from a preliminary search on PubMed. The implementation-related search string was adapted from a study of barriers and facilitators [66]. The initial search strategy was referenced against the published systematic review by Zhao et al [21] to identify supplementary terms. The search strategies used in the databases are reported in Multimedia Appendix 1. Articles were identified, screened, and selected for further review in two stages by the author: titles and abstracts, followed by the full text.

### Step 3: Study Selection

Records were selected if they involved automated patient self-scheduling. Articles were determined eligible for inclusion if they discussed the use of self-scheduling by health care organizations. Peer-reviewed articles, primary research, reviews, and original studies described in editorials in peer-reviewed journals that focused on patient self-scheduling were included. Only articles published in English were included during study selection.

For the review, the definition of self-scheduling involves real time, synchronous booking, and automated fulfillment of appointments by patients on the web or via a smartphone app for themselves. Self-scheduling does not include an appointment by a physician on behalf of a patient, as in the case of a primary care physician scheduling an appointment with a specialist for the patient. Furthermore, the definition excludes asynchronous scheduling transactions that feature the patient initiating a request for an appointment but not booking it automatically, or the slot being appointed automatically through a waitlist feature [67] or a reschedule option [68]. Patients scheduled as research participants were excluded. The definition excludes self-scheduling of providers and staff.

### Step 4: Charting the Data

A data extraction Microsoft Excel spreadsheet was developed to systematically record the details of the articles. Charted data (Multimedia Appendix 2 [5, 18, 19, 21-24, 26, 28, 29, 31-37, 42-45, 47, 48, 69-75]) included article characteristics (author, year, and country), intervention characteristics (stand-alone or component, source, introduction, description of design, and identified need), research design, setting, intervention measures assessing the impact of self-scheduling, and main results. Relevant results were extracted from the results section of each article.

### Step 5: Collating, Summarizing, and Reporting the Results

The scoping review was organized and presented in alignment with the Consolidated Framework for Implementation Research (CFIR). The conceptual framework provides guidance for the research by constructing a standard, evidence-based path for identifying, organizing, and communicating the dimensions of barriers and facilitators across organizations to advance the opportunity for adoption of the study’s findings. The framework is comprehensive, synthesizing essential constructs from 29 organizational and implementation science theories. Standard terminology promotes generalizability across disciplines (Textbox 1) [76].

Thematic analysis was performed to convey the main findings of the material.

**Consolidated Framework for Implementation Research domains and constructs.**

**Intervention characteristics**

- Intervention source
- Evidence strength and quality
- Relative advantage
- Adaptability
- Trialability
- Complexity
- Design quality and packaging
- Cost

**Outer setting**

- Patient needs and resources
- Cosmopolitanism
- Peer pressure
- External policy and incentives

**Inner setting**

- Structural characteristics
- Networks and communications
- Culture
- Implementation climate
- Readiness for implementation

**Characteristics of individuals**

- Knowledge and beliefs about the intervention
- Self-efficacy
- Individual stage of change
- Individual identification with organization
- Other personal attributes

**Process**

- Planning
- Engaging
- Executing
- Reflecting and evaluating

## Results

### Overview

**Figure 1:**
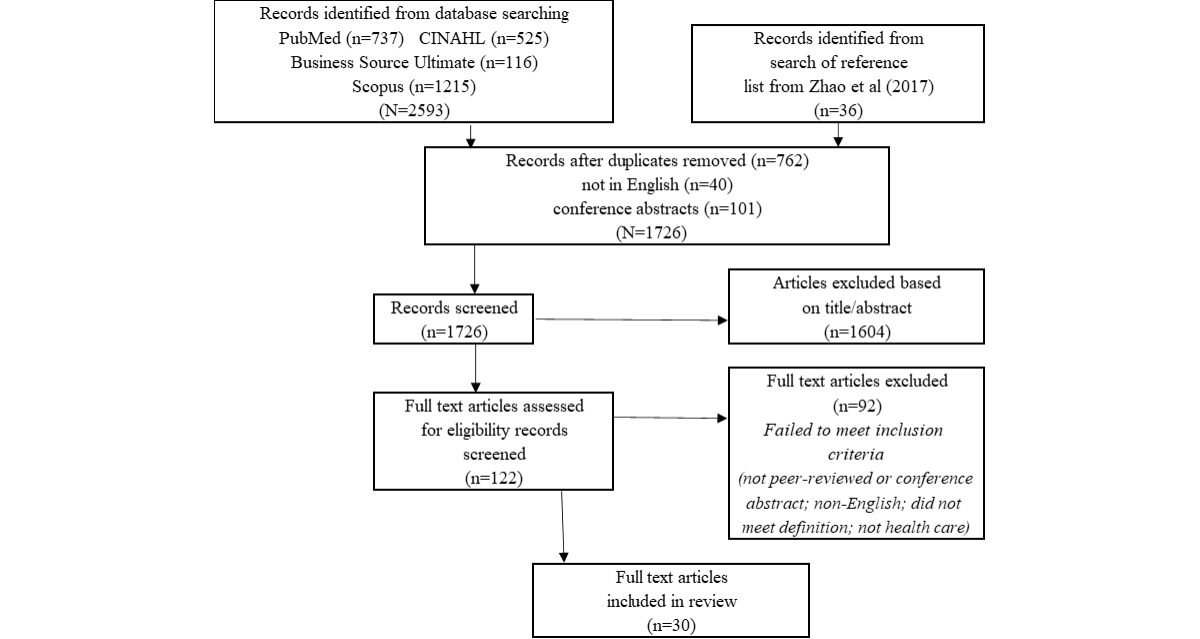
PRISMA (Preferred Reporting Items for Systematic Reviews and Meta-Analyses) diagram.

Titles and abstracts were reviewed for 1726 records, with 1604 (92.93%) records being excluded. The full texts of 7.06% (122/1726) of articles were retrieved and reviewed. In total, 5.33% (92/1726) of studies were excluded because they failed to meet the inclusion criteria. A total of 1.73% (30/1726) of studies were included in this scoping review. Figure 1 outlines the selection methodology using a PRISMA (Preferred Reporting Items for Systematic Reviews and Meta-Analyses) diagram.

The countries covered in the review include the United States [18,28,29,31,33,35,37,43,73,74], Taiwan [22,23,42], England [19,34,69,72], China [24,48,75], Australia [45,70,71], Canada [36], Iran [5,32,47], and the Philippines [26]. Another article included 7 countries in Europe [44]. Table 1 presents the countries and the number of articles from each. The first article retrieved for the scoping study was published in 2004 [18], with ≤3 articles each year up to and including 2019. In 2020, 8 articles [22,26,28,29,31,37,72,73] featuring barriers to and facilitators of automated self-scheduling were published. Table 2 displays the number of articles published by year of publication.

**Table 1:** Country-wise number of articles published (N=30).

Country	Articles, n (%)
United States	10 (33)
England	4 (13)
Taiwan	3 (10)
China	3 (10)
Australia	3 (10)
Iran	3 (10)
Canada	1 (3)
Philippines	1 (3)
7 countries in Europe	1 (3)
Other (review)	1 (3)

**Table 2:** Articles by year of publication (N=30).

Year	Articles, n (%)
2004	1 (3)
2005	0 (0)
2006	0 (0)
2007	1 (3)
2008	1 (3)
2009	1 (3)
2010	2 (7)
2011	2 (7)
2012	1 (3)
2013	2 (7)
2014	3 (10)
2015	1 (3)
2016	0 (0)
2017	2 (7)
2018	3 (10)
2019	2 (7)
2020	8 (26)

### Intervention Characteristics

#### Intervention Source

Of the 30 articles selected, 4 (13%) articles reported internal solutions for self-scheduling [28,31,37,73]. In addition to these studies, 7% (2/30) of articles were included that were published with a combination of internal and external resources [18,19]. From the 30 articles, 6 (20%) articles featured externally created interventions, 4 (13%) of which were created by a third party [35,36,43,72], 1 (3%) by the first author [71], and 1 (3%) by an unknown source [34]. The remaining articles did not elucidate the source of the intervention [5,22-24,26,32,42,44,45,47,48,70], did not feature a specific source [29,33,69,74,75], or represented a systematic review [21].

In total, of the 30 articles, 9 (30%) [18,19,22,26,32,34,45,48,72] provided some level of description of the intervention, with 4 (13%) providing only limited characteristics [22,26,34,72]. Most articles [5,18,23,24,26,32-37,43,45,47,48,70,71,73] featured the self-scheduling intervention as a stand-alone service, with a minority [19,22,28,29,31,42,44,72,74-76] including self-scheduling as a component of a larger technology offering. A systematic review [21] discussed self-scheduling in both contexts. The literature includes limited information regarding the source of the intervention. Sources were not cited as a barrier to or facilitator of implementation. This is evidenced by the volume of unknown and undescribed sources. The internally developed solutions, all reported in 2020, may imply that there is easier access for health care organizations to implement self-scheduling solutions.

#### Evidence Strength and Quality

The measurement of outcomes was a prominent element of the articles; however, the strength and quality of evidence was not presented as a determinant in the implementation of self-scheduling by the organization. The systematic review concluded that researchers demonstrated a reduced no-show rate, decreased staff labor, decreased waiting time, and improved patient satisfaction [21]. In the literature, evidence has not been measured on a consistent basis. For example, a case study documented a specific reduction in costs: a decrease of 25% of staff dedicated to scheduling, with an annual savings of US $170,000 for the organization [18]. The specifics of the roles of those personnel, their compensation, or other factors were not reported. Another study [72] reported on the intervention’s *anticipated* results. The literature did not provide a robust body of evidence that may have influenced the implementation of self-scheduling by health care organizations.

#### Relative Advantage

The advantages of the intervention compared with alternative solutions have been discussed in the literature. The comparison was made with the option of using a telephone to schedule an appointment [18,19,28,31,33,37,45,69,70,72-75]. The literature revealed the relative advantage of self-scheduling being the use of the solution at any hour to overcome patient barriers to scheduling appointments [69,72]. The findings reported that 34% [45], 46% [37], and 51% [19] of appointments were self-scheduled outside of office hours. After-hours access to the health care organization allowed early morning appointments to be filled, thus benefiting the organization [33]. In their findings, studies detailed an improved use of staff resources [18,28,37,45,70,73,75] and time savings for the patient [19,31,74]. Volk et al [28] hypothesized that self-scheduling offered patients an enhanced sense of anonymity and a diminished sense of responsibility, compared with the traditional telephone-based scheduling process.

#### Adaptability and Trialability

Faced with a surge in patient demand owing to the COVID-19 pandemic, an organization rapidly introduced the intervention [31]. This implementation provided evidence of adaptability and trialability as determinants that promoted the implementation of self-scheduling. The importance of allowing each practice the latitude to adopt their own strategy for marketing the intervention was observed; in 1 health care organization, by the second year of adoption, 20% of all slots were booked via self-scheduling [36]. Without any promotion, researchers observed a 300% increase in self-scheduled appointments within months [19]. The rapidity of implementation, customization of the solution, and patient use without promotion provide evidence of the determinants of adaptability and trialability to facilitate implementation.

#### Complexity

Although most of the studies did not describe the intervention, several studies made note of elements that revealed the complexity of the intervention. Slot unavailability was cited as a deterrent for patients attempting to self-schedule [36,45]. Ease of use was confirmed to be a key attribute for self-scheduling from the perspective of the patient [23,29]. These findings contrast those of Lee et al [22], who concluded that ease of use was not a facilitating attribute; instead, the researchers ascertained that performance expectancy was the determinant. Solutions that were bundled with triage featured an algorithm that diverted patients with acute symptoms from the self-scheduling option [19,31]. In all, 3% (1/30) of organizations reviewed appointments manually for safety and appropriateness [73]. The complexity of the intervention was reported to be important to manage [33], suggesting that it is a determinant of implementation success for health care organizations.

#### Design Quality and Packaging

The literature did not elaborate on the design quality and packaging of the intervention, except for sample screenshots of the patient interface [18,32,69]. Studies have highlighted the importance of integration with other information technology systems [33,36]. In all, 3% (1/30) of studies pointed out a predetermined lack of publicity: a health care organization during the pandemic avoided promotion to prevent artificially inducing additional patient demand [31]. A key factor in adoption was the organization making patients aware of the intervention [36,45,70]. Brochures made available to patients were reported to be ineffective in raising awareness [36,70,71]. Health care organizations documented the importance of presenting self-scheduling to patients using communication methods planned locally, as varying methods of approach may affect outcomes [72-74].

#### Cost

Although concern about cost was revealed as a barrier to physicians’ interest in offering self-scheduling, information about the cost of the intervention to the health care organization was not addressed in the literature [21]. One author funded the intervention personally [18]. However, no details were provided regarding the amount spent for the intervention.

### Outer Setting

#### Patient Needs and Resources

Concerns have been raised regarding possible disparities in care access for Medicaid recipients in the United States owing to lower provider count and longer distance to appointments via third-party self-scheduling platforms [43], as well as lower use rates of self-scheduling compared with non-Medicaid patients [31,73]. Research has provided evidence of diminished access to self-scheduling for rural patients compared with urban patients [35]. Low socioeconomic status was a driver of low adoption rates [45,72], with younger [19,37,45,72,73] women [19,37] who were employed [45] and patients with higher education [24,45] using the self-scheduling platform. Younger patients expressed the value of self-scheduling, as compared with users more senior to them [44,48,74]. One study [34] concluded that older patients were higher users; their study focused on the self-scheduling of specialty visits following a primary care physician’s referral, thereby indicating that patients were specifically instructed to self-schedule. Patients with comorbidities were shown to be more frequent users than other patients [73]. Although most studies measured patient awareness, characteristics, use, and intention to use, there has been a growing interest over time in accounting for patients’ needs and resources.

Multiple studies identified patients’ access to the internet and computers as a potential barrier to the use of self-scheduling [35,45,70,71,74]. In a postintervention focus group, Mendoza et al [26] confirmed stakeholders’ concerns regarding access to the internet, noting that a barrier may be internet speed, in that a desired slot may be taken by another patient if the bandwidth is inadequate. In a systematic review, Zhao et al [21] concluded that patients’ reluctance to adopt self-scheduling results from prior experience with the internet and computers, as well as preferences for communication methods. Addressing people’s trust to enhance use is essential [29]. Researchers have identified gaps between people’s interest in the technology and its use [29,44], and awareness of the technology and its use [71,75].

Cosmopolitanism—the extent to which an organization is networked with others external to itself—and peer pressure have not been discussed in the literature.

#### External Policy and Incentives

Research was influenced by government policies in several studies: a federally funded initiative was established to fast-track the advancement of health information technologies across Canada [36]. The British government recommended the *novel use of information technology* to meet government-mandated targets for appointment offerings [19]. The *Choose and Book System* studied by Parmar et al [34] was the national electronic referral and booking service introduced in England in 2004 which has since been replaced. Studies by researchers from China described the web-based appointment system, the use of which, as of 2009, has been supported by the Ministry of Health for deployment by all hospitals [24,48]. In Australia, the National E-Health Strategy incorporated electronic communication between patients and providers [45]. Iran mandated that hospitals offer self-scheduling for outpatients, although compliance has been limited [47].

In their multinational research in Europe, Santana et al [44] acknowledged the importance of the prevailing legal and regulatory environment of each nation, as well as a country’s health care policies and technological advances, in the adoption of self-scheduling. The influence of external policy and incentives at the national level on all aspects of eHealth have been scrutinized by researchers worldwide [77].

In addition to the impact of the government, other external factors may play a role in the uptake of self-scheduling including the COVID-19 pandemic [31].

### Inner Setting

The key elements of the structural characteristics of the research settings are included in Multimedia Appendix 2. Of the 28 studies that defined the research setting, 14 (50%) were based in outpatient practices [5,18,19,32,34-37,43,45,70-72,74], 10 (36%) were based in medical centers [22, 24, 26, 28, 31, 42, 47, 48, 73, 75], and 4 (14%) surveyed community members [23,29,44,69]. Among the outpatient practice studies, 13% (4/30) featured settings of single specialties: 7% (2/30) dermatology [35,37], 3% (1/30) audiology [34], and 3% (1/30) genitourinary [19].

Data were not included in the studies for networks and communication or culture. Limited information was provided about the implementation climate. Friedman [18] conveyed that his physician colleagues “turned white as ghosts” at the suggestion of implementing self-scheduling, citing concerns about transparency; however, most adopted the platform. Acknowledging reluctance, Craig [33] advised, “like anything new, [self-scheduling] will take some getting used to.”

Habibi et al [5] determined the importance of rendering favorable services owing to *increased competition*. This study was joined by 9 others that expressed the priority for change [18,22,24,26,28,31,42,45,47]. The sense of urgency increased over time. Zhang et al [24] reported lines forming late at night and “incidents of knife attacks at hospitals” resulting from patients’ frustrations.

The importance of problem solving in the outpatient environment, which is the face of the hospital, was emphasized [26]. Lee et al [22] concluded that the impression of service quality put forth by the self-scheduling technology was a key success factor for a hospital to “gain an...advantage...in an increasingly competitive healthcare market.” Volk et al [28] described the current environment that led to the introduction of the intervention as “threatening the organization's reputation and financial well-being.”

Readiness for implementation was not addressed in detail: 3% (1/30) of studies [32] mentioned about providing the secretaries with a tablet and training; however, no other study described the engagement of leadership, available resources, or access to knowledge and information.

### Characteristics of Individuals

#### Overview

Limited information in the body of literature included in this study was provided about individuals engaged in self-scheduling. In all, 3% (1/30) of studies described the hesitancy of physicians, although a revision to the intervention (pop-up menus) was developed during the project to address it [36]. Habibi et al [32] reflected on the “interest and eagerness of physicians,” which contributed to the success of the self-scheduling intervention. The other articles in the scoping study offered little insight into the characteristics of the individuals participating in the intervention and whether individuals served as barriers to or facilitators of adoption.

#### Process

Limited information was provided about the process associated with the intervention: planning, engaging, executing, and reflecting and evaluating. Of the 30 studies, 1 (3%) study [36] elaborated on the importance of managing the physicians’ expectations about slot availability, as patients may lose interest and discontinue the use of the system based on insufficient slots. In all, 7% (2/30) of studies postulated the importance of integrating the self-scheduling platform with the electronic medical record system [33,36]. Volk et al [28] documented a leadership task force. The literature offers limited insights into the implementation process.

## Discussion

### Existing Knowledge

This scoping review located 30 published articles that described synchronous, automated self-scheduling tools for patient appointments. The number of studies related to self-scheduling increased over time. The growing volume of research reflects the popularity of the technology, signaling its broadening appeal. Research performed in the same community-based clinic setting concluded a low intention to use [45,70,71]. However, low intention to use was not demonstrated in a study since 2015, perhaps reflecting the now pervasive use of computers. Patients’ trust in the intervention has been studied as a possible barrier to the intervention [29]. Studies have continued to identify gaps between the interest and awareness of the technology and its use [29,75]. Researchers have concluded that concerns about access to the internet persist [26]. The introduction of self-scheduling in the context of a hospital as a business entity with financial interests commenced in 2020, perhaps reflecting the opportunity that a self-scheduling offering is no longer considered an initiative to appeal to innovators but rather a necessity of service delivery. Lee et al [22] determined that ease of use was no longer a factor of patients’ continuous use, concluding that the system is now “stable, reliable, and well designed.” This study reflected patients’ increasing comfort with technology, which is supported by the literature about other consumer-oriented offerings such as telemedicine [78,79]. Articles aimed at optimization methods for scheduling, such as recommendations for demand matching [80], were formulated on a platform of automated scheduling, a reflection that the literature has evolved from the foundational elements of implementation to a more sophisticated approach.

Efforts to determine the effect of self-scheduling may be hindered by the incorporation of the intervention as an element in a suite of technologies. Of the 30 studies, 11 (37%) studies in this scoping review [19,22,28,29,31,42,44,69,72,74,75] included self-scheduling as a component of a larger technology initiative, which may indicate that another intervention that was aligned with self-scheduling was the source of the organizational benefit.

The scoping study incorporated a systematic review that was conducted in 2017. The systematic review [21] reported the advantages of self-scheduling for organizations. In the literature before the systematic review, most gains were reported to have the potential to benefit the organization. Beginning in 2017, the advantages of self-scheduling have increasingly focused on the outer setting. Organizations react to consumers’ access to technology and their competitive environment. Furthermore, the benefits of self-scheduling from the patients’ perspective—satisfaction, time, convenience, and engagement—were increasingly referred to as potential rewards. Table 3 highlights the changes in the focus of the literature related to the identified need for the intervention. This may reflect an alteration in the determinants of adoption.

In a systematic review, Zhao et al [21] concluded that cost, flexibility, safety, and integrity were the barriers to adoption. Except for safety, these organizational barriers have not been replicated in the literature since 2017 [26,73]. However, the research upon which these conclusions were based drew upon the popular literature except for a 2004 case study [18] and a 2007 commentary [33], both of which noted providers’ hesitancy. The lack of evidence-based organizational barriers over time may mean that the obstacles have historically been organizations’ perceptions of patient behavior. The reluctance of patients to adopt based on their experience with computers reported in the systematic review [21] was not reproduced other than the potential impact of broadband speed noted in a focus group [26]. Despite the lack of evidence-based barriers, use of self-scheduling has continued to be reported at low rates during the period of 2017-2020 [47,72,75].

**Table 3:** Identified need for self-scheduled based on literature mentions.

Identified need			Mentions, n (%)		
			Before 2017 (n=23)		2017-2020 (n=25)
**Inner setting**			**14 (61)**		**9 (36)**
	Organization’s cost and labor	4 (17) [18,23,45,70]		5 (20) [28,29,37,73,75]	
	Organization’s resource use (no-shows)	6 (26) [33-36,42,45]		3 (12) [29,37,73]	
	Organization’s communication and information transparency	2 (9) [23,44]		1 (4) [22]	
	Alternative to organization’s existing scheduling method	2 (9) [19,74]		0 (0)	
**Outer setting**			**9 (39)**		**16 (64)**
	Consumer access to technology	1 (4) [71]		1 (4) [32]	
	Organization’s need to compete	1 (4) [42]		1 (4) [5]	
	Government policy	1 (4) [19]		1 (4) [47]	
	Patient satisfaction	1 (4) [42]		4 (16) [26,29,37,47]	
	Patient convenience	2 (9) [70,74]		5 (20) [31,43,69,72,73]	
	Patient wait time	3 (13) [23,24,48]		3 (12) [29,32,37]	
	Patient engagement	0 (0)		1 (4) [29]	

### Opportunities for Research

Self-scheduling may offer value to health care organizations. Additional research regarding the barriers to and facilitators of implementation is warranted.

#### Nomenclature

The terminology used to describe self-scheduling presented a challenge for the scoping study. The function—*scheduling*—was documented using a variety of labels, leading to a diversity of terms for the intervention under study. Standard terminology was not present in the research findings: the US-based research incorporated insurance coverage, lacking direct comparison with the non–US-based research that incorporated findings about *social grade* [71,72] and *socioeconomic status* [45]. Other characteristics, such as age range, varied in reporting. The lack of a standard vocabulary for the intervention and its users, uptake, evidence, and so forth has implications for research, as well as acceptance and adoption by health care organizations. This may present a barrier to organizations seeking knowledge about self-scheduling. Authors should incorporate keywords that reflect both breadth and depth to boost identification [81].

#### Implementation Framework

Within the CFIR, much of the research to date has focused on the intervention characteristics of self-scheduling, including the intervention source, relative advantage, adaptability, trialability, complexity, and design quality and packaging. The characteristics are largely presented as effects of the intervention, not the determinants of implementation. Evidence strength and quality may be enhanced through improved research methods. The discussion of the cost of the intervention and its ongoing maintenance is limited. There is no consistent approach to the study of the intervention’s characteristics to inform adoption. After presenting the results of a pilot study, researchers in 2020 [37] concluded the following:

We hope to encourage other colleagues to explore and share their experiences...and to stimulate conversation regarding implementation of technology to improve access to care.

This request may signal a current gap in the literature regarding barriers to and facilitators of the implementation of self-scheduling.

Concepts warranting further research include the inner setting and individual characteristics contained in the CFIR. Qualitative research is needed to provide context and understanding of why health care organizations face barriers to successful outcomes identified by quantitative surveys. These may be present in the inner setting of organizations and individuals’ characteristics, constructs that are largely unexplored by research on self-scheduling.

Although there is no consistent definition or inclusion of characteristics, within the outer setting, patient needs and resources in the form of gender, race, socioeconomic status, education level, employment, geography, computer access, experience, and literacy were explored by researchers. The nonstandard approach makes it difficult to determine the barriers to and facilitators of health care organizations to meet patients’ needs. For example, rural populations face more problems in accessing care [82,83]. Consideration may be given to customized interventions for vulnerable patient populations, a topic unexplored in the literature. Otherwise, existing inequities related to the broadening gap of rural–urban disparities in life expectancy may be perpetuated [84].

External policy and incentives play a role in influencing self-scheduling, primarily at the country level. Although researchers mention the national initiatives, no details were provided about the initiative serving as a barrier or facilitator, or how that influence could be successful. Recognizing the importance of policies and regulations in health care technology [85], researchers may explore the characteristics and impact of external policies and incentives for nations that require self-scheduling to be offered by health care organizations.

#### Technology in Health Care

Researchers have explored the challenges of implementing other information and communication technologies that have exhibited evidence for improving systems, processes, and outcomes in health care. Documented inner setting obstacles to technology implementation include a culture that lacks receptivity [86], an absence of trust [87], a resistance to change [88], workflow changes that were required for uptake [89,90], and upfront and ongoing costs of the solution [91]. The Systems Engineering Initiative for Patient Safety 2.0 model was introduced to account for human factors systems, extending into the concepts of adaptation, engagement, and configuration [92]. The determinants identified by researchers evaluating the implementation of other technologies by health care organizations may offer insight into a framework to explore the limited uptake of self-scheduling.

#### Health Care Providers

Although there are references to the providers’ perspective in the academic literature incorporated in this scoping study [18,32,33,36], these have not been examined in detail. For the only study that reported measuring it, physician punctuality improved after the intervention was introduced, and the researchers surmised that the enhancement resulted from the physicians’ enthusiasm about the solution, as well as the reminder of the first appointment of the day transmitted via text from the self-scheduling tool [32]. Although 3% (1/30) of studies [26] concluded that they were able to eliminate some elements of patient dissatisfaction, the researchers determined that 40% of the dissatisfaction was a function of the physicians being late and canceling clinics, albeit the intervention they launched enabled the staff to inform patients of the delays. The connectivity of the intervention to its offering—the provider’s time—is largely unexplored.

To date, the literature on the uptake of self-scheduling has focused on the end user: patients’ awareness, characteristics, use, and intention to use. As self-scheduling platforms aim to provide a limited inventory of providers’ time, the provider is an equally important stakeholder. Further research may reveal ideas, variables, and determinants that are not yet recognized by health care organizations. The literature needs to focus more on the integration of technology into work systems. Research on providers as resisters of other automated health care administrative tools, such as telemedicine, has proliferated [93]. Similar research techniques may be applied to garner a better understanding of self-scheduling.

#### Relationships

The existing literature does not elucidate the factors that promote or impede the uptake of self-scheduling by health care organizations. The absence of aggregation and examination of barriers and facilitators may reflect the complexity of self-scheduling as an intervention. As demonstrated in the literature, the solution is influenced by the intervention’s characteristics, the outer and inner settings of the health care organization, individual stakeholders, and the process related to the intervention. Self-scheduling cannot be implemented and scaled without a comprehensive understanding of these factors. In contrast to the focus on dissecting individual components defined by the CFIR, the success of an implementation by a complex, adaptive health care organization is informed by the interdependence of the determinants [94]. The exploration of enablers and obstacles by examining the contingent and reciprocal relationships within health care organizations may better illuminate the implementation determinants for self-scheduling.

### Limitations

The author (EW) conducted the screening process, which may have introduced selection bias. The lack of a standard naming convention may have resulted in missing relevant articles for the scoping review. Given the large number of findings from countries with a primary language other than English, the inclusion of English-only articles may have missed publications that were not accessible from the databases deployed in the search strategy.

In contrast to systemic reviews, scoping studies, by definition, do not incorporate a quality assessment of individual studies; therefore, it is challenging to assess whether studies produce robust findings [64]. As such, data synthesis and interpretation are limited [63].

An agreement on common measures to identify and monitor the impact of self-scheduling is required. Research that tracked the most cited advantage of reducing the no-show rate failed to accompany the discourse with a definition of said rate.

### Conclusions

This scoping review cataloged existing knowledge and identified gaps in knowledge regarding the uptake of automated self-scheduling by health care organizations. The intervention was defined. There was evidence of the broadening appeal and demonstrable benefits of automated self-scheduling; however, the uptake remained low.

Prior research examined implementation effectiveness; this review focused on barriers to and facilitators of self-scheduling by health care organizations. Outer setting determinants to include national policy, competition, the response to patients’ needs, and technology access played an increasing role in influencing implementation over time. Automated self-scheduling may offer a solution to health care organizations striving to positively affect access.

## Appendix

Multimedia Appendix 1 Search strategy.

Multimedia Appendix 2 Charted data.

## Abbreviations

CFIR: Consolidated Framework for Implementation Research
PRISMA: Preferred Reporting Items for Systematic Reviews and Meta-Analyses
PRISMA-S: Preferred Reporting Items for Systematic Reviews and Meta-Analyses Search
