# Supplementary material for: Barriers to and Facilitators of Automated Patient Self-scheduling for Health Care Organizations: Scoping Review
Source: J Med Internet Res. 2022 Jan 11;24(1):e28323. doi: 10.2196/28323 (PMC8790681; doi:10.2196/28323)
Supplement: Multimedia Appendix 1 [file jmir_v24i1e28323_app1.docx]

**Multimedia Appendix 1.** Search strategy.

PubMed Search Strategy:

**((Patients[MESH] OR patient* [tw]) AND (((("Appointments and Schedules"[Mesh] OR schedul* [tw]) AND (online [tw] OR self-serve [tw] OR web-based [tw] OR internet-based [tw] OR self-service [tw])) OR "e-book*" [tw] OR "online appointment*" [tw] OR "online book*" [tw] OR "self-serve" OR "automated schedul*" [tw] OR "Web-based schedul*" [tw] OR "self-schedul*" [tw] OR "online schedul*" [tw] OR "e-schedul*" [tw] OR "Internet scheduling" [tw]) AND ("Evaluation Studies as Topic"[Mesh] OR Routin* [tw] OR Integrat* [tw] OR Facilitate* [tw] OR Barrier* [tw] OR Implement* [tw] OR Adopt* [tw]))) NOT ((Patients[MESH] OR patient* [tw]) AND (((("Appointments and Schedules"[Mesh] OR schedul* [tw]) AND (online [tw] OR self-serve [tw] OR web-based [tw] OR internet-based [tw] OR self-service [tw])) OR "e-book*" [tw] OR "online appointment*" [tw] OR "online book*" [tw] OR "self-serve" OR "automated schedul*" [tw] OR "Web-based schedul*" [tw] OR "self-schedul*" [tw] OR "online schedul*" [tw] OR "e-schedul*" [tw] OR "Internet scheduling" [tw]) AND ("Evaluation Studies as Topic"[Mesh] OR Routin* [tw] OR Integrat* [tw] OR Facilitate* [tw] OR Barrier* [tw] OR Implement* [tw] OR Adopt* [tw])))**

Scopus Search Strategy:

( ( TITLE-ABS-KEY ( ( patient* AND ( appointment* OR schedul* ) AND ( online OR self-serve OR web-based OR internet-based OR self-service ) ) ) ) OR ( TITLE-ABS-KEY ( ( "e-booking" OR "online appointment*" OR "online book*" OR ( "self-serve" AND schedule* ) OR "automated schedul*" OR "Web-based schedul*" OR "self-schedul*" OR "online schedul*" OR "e-schedul*" OR "Internet scheduling" ) AND ( medical OR patient* ) ) ) ) AND ( TITLE-ABS-KEY ( evaluation OR routin* OR integrat* OR facilitate* OR barrier* OR implement* OR adopt* ) )

CINAHL Search Strategy:

Interface - EBSCOhost Research Databases
Search Screen - Advanced Search
Database - CINAHL Plus with Full Text

| # | Query | Results |
| --- | --- | --- |
| S8 | S6 AND S7 | 525 |
| S7 | ( (MH "Evaluation+") OR (MH "Health Services Accessibility+") ) OR ( Routin* OR Integrat* OR Facilitate* OR Barrier* OR Implement* OR Adopt* ) | 727,197 |
| S6 | S4 OR S5 | 1,850 |
| S5 | "e-book*" OR "online appointment*" OR "online book*" OR "self-serve" OR "automated schedul*" [tw] OR "Web-based schedul*" [tw] OR "self-schedul*" [tw] OR "online schedul*" OR "e-schedul*" OR "Internet scheduling" | 983 |
| S4 | S1 AND S2 AND S3 | 898 |
| S3 | (online OR self-serve OR web-based OR internet-based OR self-service | 92,651 |
| S2 | (MH "Patients+") OR ( patient OR patients ) | 2,170,278 |
| S1 | ( (MH "Appointments and Schedules+") ) OR schedul* | 99,581 |

Business Source Ultimate Search Strategy:

Interface - EBSCOhost Research Databases
Search Screen - Advanced Search
Database - Business Source Ultimate

| # | Query | Results |
| --- | --- | --- |
| S12 | S8 AND S11 | 116 |
| S11 | S9 OR S10 | 1,702,363 |
| S10 | Evaluation OR Routin* OR Integrat* OR Facilitate* OR Barrier* OR Implement* OR Adopt* | 1,601,811 |
| S9 | (DE "PROJECT evaluation") OR (DE "RESEARCH" OR DE "EMPIRICAL research" OR DE "FEASIBILITY studies" OR DE "FIELD work (Research)" OR DE "OPERATIONS research" OR DE "QUALITATIVE research" OR DE "QUANTITATIVE research" OR DE "RESEARCH & development") | 134,461 |
| S8 | S6 OR S7 | 470 |
| S7 | S1 AND S2 AND S3 | 260 |
| S6 | S4 AND S5 | 263 |
| S5 | patient* OR medical | 873,418 |
| S4 | "e-booking" OR "online appointment*" OR "online book*" OR "self-serve" OR "automated schedul*" OR "Web-based schedul*" OR "self-schedul*" OR "online schedul*" OR "e-schedul*" OR "Internet scheduling" | 11,600 |
| S3 | online OR self-serve OR web-based OR internet-based OR self-service | 982,523 |
| S2 | patient* | 215,814 |
| S1 | DE "SCHEDULING" OR ( appointment* OR schedul* ) | 536,906 |
